# First COVID-19 infections in the Philippines: a case report

**DOI:** 10.1186/s41182-020-00203-0

**Published:** 2020-04-14

**Authors:** Edna M. Edrada, Edmundo B. Lopez, Jose Benito Villarama, Eumelia P. Salva Villarama, Bren F. Dagoc, Chris Smith, Ana Ria Sayo, Jeffrey A. Verona, Jamie Trifalgar-Arches, Jezreel Lazaro, Ellen Grace M. Balinas, Elizabeth Freda O. Telan, Lynsil Roy, Myvie Galon, Carl Hill N. Florida, Tatsuya Ukawa, Annavi Marie G. Villaneuva, Nobuo Saito, Jean Raphael Nepomuceno, Koya Ariyoshi, Celia Carlos, Amalea Dulcene Nicolasora, Rontgene M. Solante

**Affiliations:** 1 San Lazaro Hospital, Manila, Philippines; 2 School of Tropical Medicine and Global Health, Nagasaki University, Nagasaki, Japan; 3 Faculty of Infectious and Tropical Diseases, London School of Hygiene and Tropical Medicine, London, UK; 4 Department of Microbiology, Faculty of Medicine, Oita University, Oita, Japan; 5 Institute of Tropical Medicine, Nagasaki University, Nagasaki, Japan; 6 Research Institute for Tropical Medicine, Alabang, Philippines

**Keywords:** Case report, COVID-19, SARS-CoV-2, Coronavirus, Philippines, Manila

## Abstract

**Background:**

The novel coronavirus (COVID-19) is responsible for more fatalities than the SARS coronavirus, despite being in the initial stage of a global pandemic. The first suspected case in the Philippines was investigated on January 22, 2020, and 633 suspected cases were reported as of March 1. We describe the clinical and epidemiological aspects of the first two confirmed COVID-19 cases in the Philippines, both admitted to the national infectious disease referral hospital in Manila.

**Case presentation:**

Both patients were previously healthy Chinese nationals on vacation in the Philippines travelling as a couple during January 2020. Patient 1, a 39-year-old female, had symptoms of cough and sore throat and was admitted to San Lazaro Hospital in Manila on January 25. Physical examination was unremarkable. *Influenza B*, human coronavirus 229E, *Staphylococcus aureus* and *Klebsiella pneumoniae* were detected by PCR on initial nasopharyngeal/oropharyngeal (NPS/OPS) swabs. On January 30, SARS-CoV-2 viral RNA was reported to be detected by PCR on the initial swabs and she was identified as the first confirmed COVID-19 case in the Philippines. Her symptoms resolved, and she was discharged. Patient 2, a 44-year-old male, had symptoms of fever, cough, and chills. *Influenza B* and *Streptococcus pneumoniae* were detected by PCR on initial NPS/OPS swabs. He was treated for community-acquired pneumonia with intravenous antibiotics, but his condition deteriorated and he required intubation. On January 31, SARS-CoV-2 viral RNA was reported to be detected by PCR on the initial swabs, and he was identified as the 2nd confirmed COVID-19 infection in the Philippines. On February 1, the patient’s condition deteriorated, and following a cardiac arrest, it was not possible to revive him. He was thus confirmed as the first COVID-19 death outside of China.

**Conclusions:**

This case report highlights several important clinical and public health issues. Despite both patients being young adults with no significant past medical history, they had very different clinical courses, illustrating how COVID-19 can present with a wide spectrum of disease. As of March 1, there have been three confirmed COVID-19 cases in the Philippines. Continued vigilance is required to identify new cases.

## Background

The novel coronavirus 2019 (COVID-19) is responsible for more fatalities than the severe acute respiratory syndrome (SARS) coronavirus, despite being in the initial stage of a global pandemic. It is thought that the index case occurred on December 8, 2019, in Wuhan, China [1]. Since then, cases have been exported to other Chinese cities, as well as internationally, highlighting concern of a global outbreak [2]. The first suspected case in the Philippines was investigated on January 22, 2020, and 633 suspected cases have been reported as of March 1. Of them, 183 were in the National Capital Region of Manila, of whom many were admitted to San Lazaro Hospital (SLH) in Manila, the national infectious disease referral hospital [3, 4]. We describe the epidemiologic and clinical characteristics of the first two confirmed COVID-19 cases in the Philippines, including the first death outside China.

## Case presentation

**Fig. 1:**
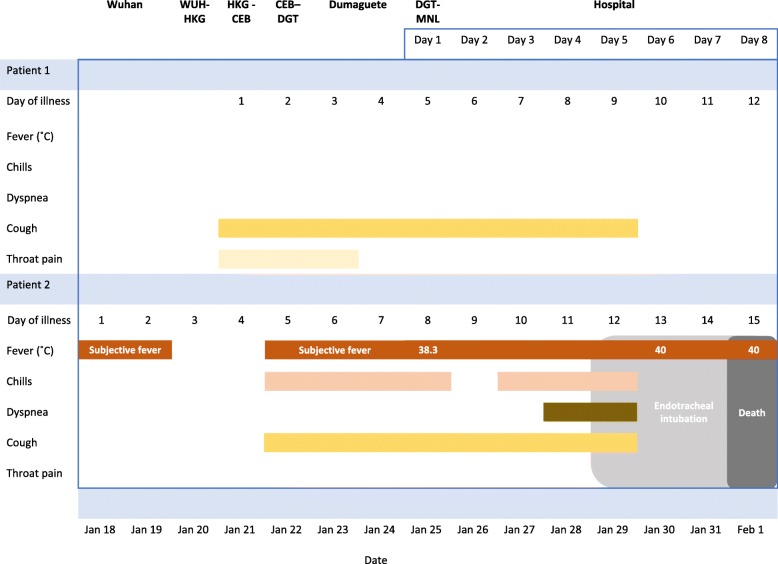
Timeline of symptoms according to day of illness and day of hospitalisation

In this case report, we describe two cases: patient 1, the first confirmed COVID-19 case, and patient 2, the second confirmed case, even though the symptoms of patient 2 started first. The cases are presented based on reports from the clinicians involved in patient care and results of investigations available to them at the time. Figure 1 shows a timeline of symptoms for both patients according to the day of illness and day of hospitalisation.

## History prior to hospitalisation

**Fig. 2:**
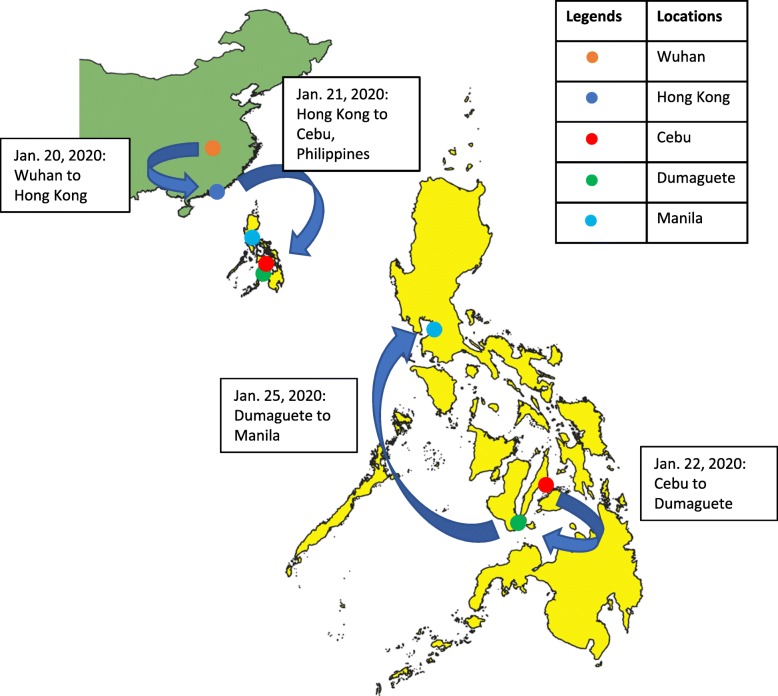
Travels of patient 1 and 2

Both patients were Chinese nationals on vacation in the Philippines travelling as a couple. They had no known comorbidities and reported no history of smoking. Patient 2, a 44-year-old male, reported fever on January 18, 2020, whilst the couple were residing in Wuhan, China. It was reported that he was in contact with someone that was unwell in Wuhan, but not that he had visited the seafood market. During January 20 to 25, they travelled from Wuhan via Hong Kong to several locations in the Philippines (Fig. 2). Patient 1, a 39-year-old female, developed cough and sore throat on January 21. Due to persistence of symptoms of patient 2, they travelled to Manila on January 25. In Manila, patient 2 was denied entry to a hotel because he was febrile and both patients were transferred to San Lazaro Hospital (SLH), the national referral hospital for infectious diseases [4]. On admission, patient 2 was classified as a COVID-19 person under investigation (PUI) based on his travel history and fever [2] and was transferred to a designated isolation area with negative pressure rooms. Patient 1 did not fit the PUI criteria due to absence of fever, but was also isolated because of possible exposure.

## Clinical course of patient 1

**Fig. 3:**
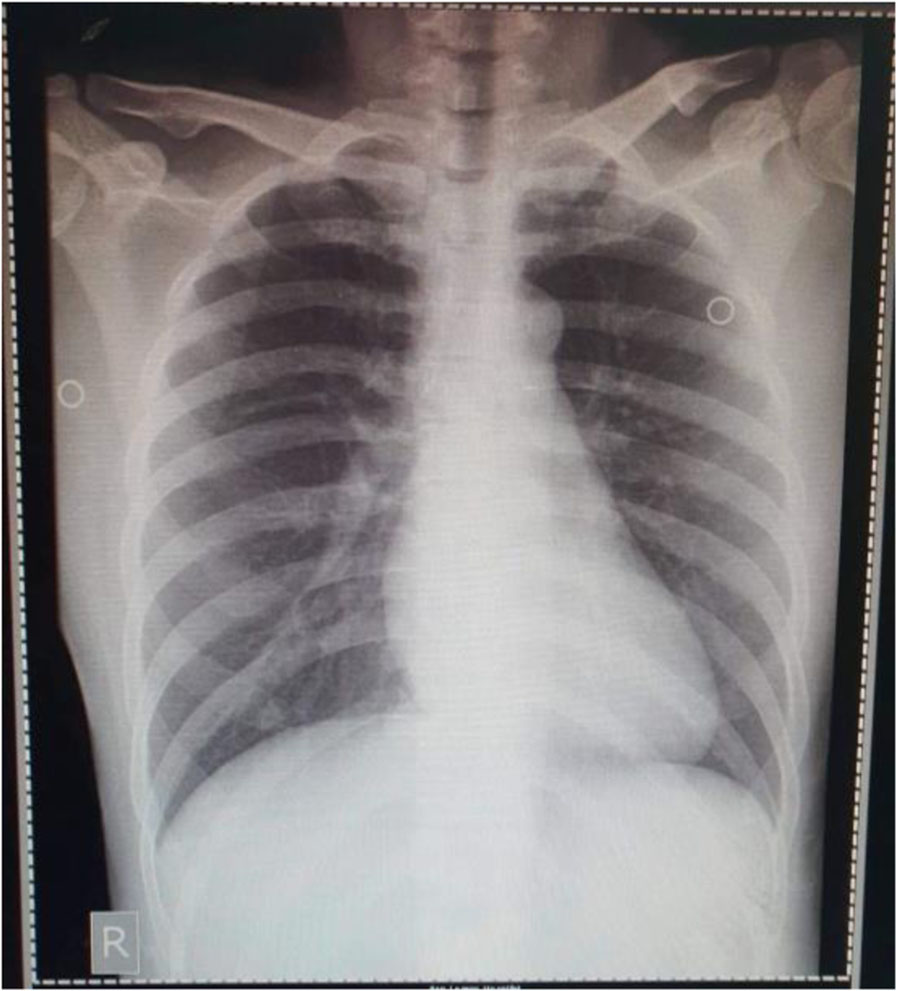
Posteroanterior chest radiograph of patient 1, 27 January 2020 (illness day 7). Unremarkable

On admission to the ward on January 25 (illness day 5), patient 1 complained of a dry cough, but the sore throat had improved. She was awake and conversant with a blood pressure of 110/80, HR 84, RR 18 and temperature 36.8 °C. Her chest was clear. The remainder of the physical examination was unremarkable. Nasopharyngeal and oropharyngeal swab (NPS/ORS) specimens were collected and sent to the Research Institute for Tropical Medicine (RITM) in Muntinlupa City [5]. A chest radiograph was reported as unremarkable (Fig. 3).

On January 27, the results were released of a commercially available respiratory pathogen multiplex real-time PCR for detection of pathogen genes on the NPS/OPS samples (FTD Respiratory pathogens 33, Fast Track Diagnostics) at the RITM Molecular Biology Laboratory. These assays reported detection of *Influenza B* viral RNA, human coronavirus 229E viral RNA, *Staphylococcus aureus* DNA and *Klebsiella pneumoniae* DNA. A 10-day course of oseltamivir 75 mg BID was given on the basis of the influenza result. The NPS/OPS specimen was then sent by RITM to the Victorian Infectious Disease Reference Laboratory (VIDRL) in Melbourne, Australia, for COVID-19 testing [6].

On January 29, further NPS/ORS specimens were collected and sent to the RITM. On January 30, the result of the initial NPS/OPS sent to VIDRL reported detection of 2019-nCoV (subsequently termed SARS-CoV-2) viral RNA by real-time PCR. The patient was thus identified by the Department of Health as the first confirmed COVID-19 case in the Philippines [6].

On illness days 6 to 10, she remained afebrile with minimal cough and clear breath sounds. During this time, real-time PCR for detecting SARS-CoV-2 was established at the RITM using the Corman et al. protocol [7]. Further NPS/OPS specimens collected on January 29 (reported on January 31) and January 31 (reported on February 2) also reported detection of SARS-CoV-2 viral RNA. On illness day 11, the patient reported resolution of symptoms. She remained afebrile and clinically stable apart from two episodes of loose watery stool on illness day 12. Further samples were collected on February 2 and 4. On February 8 (illness day 19), she was discharged when SARS-CoV-2 was no longer detected on an NPS/OPS sample.

## Clinical course of patient 2

In contrast, patient 2 experienced a more severe clinical course. On admission (illness day 8), he reported fever, cough and chills. On examination, he was awake and conversant with a temperature of 38.3 °C, blood pressure of 110/80, HR 84, RR 18, and SpO_2_ of 96% on room air. His chest was clear. The remainder of the physical examination was unremarkable.

A working diagnosis of community-acquired pneumonia and COVID-19 suspect was made. He was started on ceftriaxone 2 g intravenously (IV) once daily (OD) and azithromycin 500 mg OD. NPS/ORS specimens were collected and sent to the RITM. On January 27, the results of a respiratory pathogen real-time PCR detection panel performed at RITM on the NPS/OPS samples were released, reporting detection of *Influenza B* viral RNA and *Streptococcus pneumoniae* DNA. The NPS/OPS samples were sent to the VIDRL for additional testing. Oseltamivir 75 mg BID was commenced on the basis of the influenza result.

**Fig. 4:**
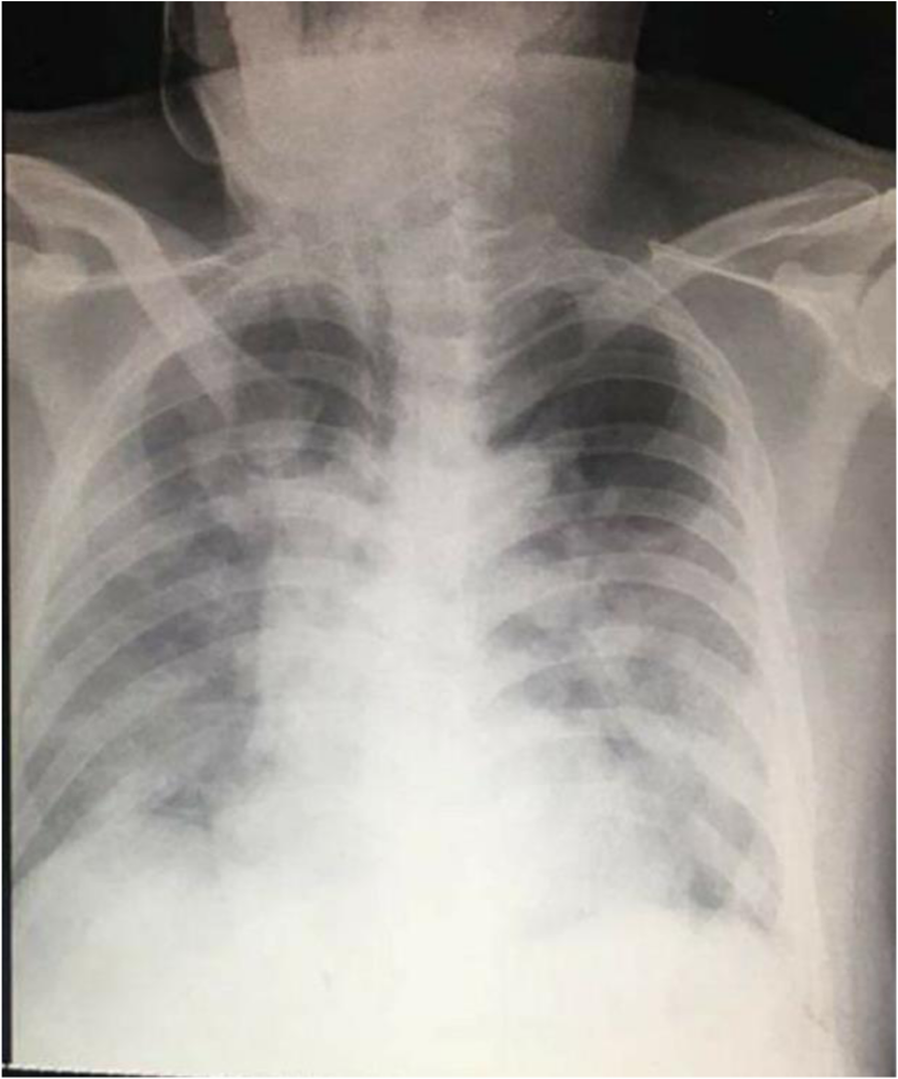
Posteroanterior chest radiograph of patient 2, 27 January 2020 (illness day 10). Hazy infiltrates in both lung fields consistent with pneumonia

During illness days 9 and 10, his fever continued with occasional non-productive cough. He remained clinically stable apart from intermittent SpO_2_ desaturations of 93–97% on 2–3 L/min of oxygen. On illness day 11, he developed increasing dyspnoea with reduced SpO_2_ at 88% despite 8 L/min of oxygen via a face mask and haemoptysis and was noted to have bilateral chest crepitations. A chest radiograph was reported as showing hazy infiltrates in both lung fields consistent with pneumonia (Fig. 4). Meropenem 2 g IV three times a day (TDS) was commenced.

**Fig. 5:**
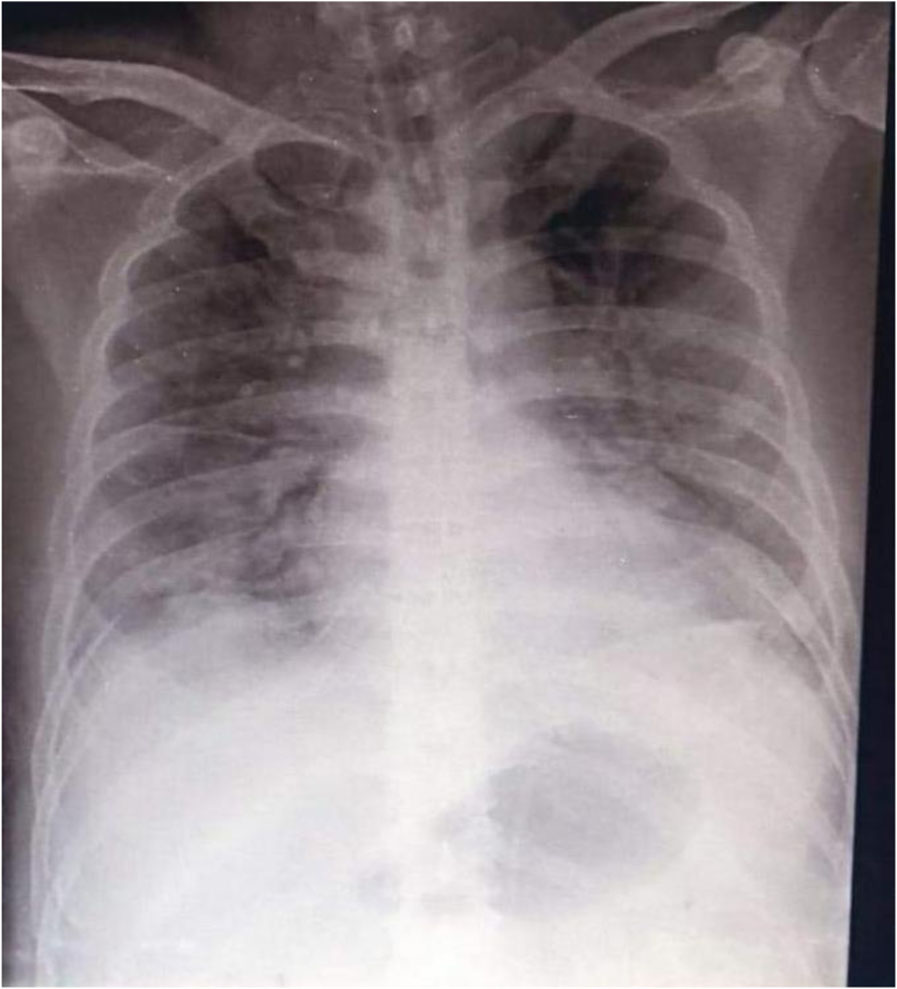
Posteroanterior chest radiograph of patient 2, 30 January 2020 (illness day 13). Endotracheal tube in situ approximately 2 cm above the carina. There is worsening of the previously noted pneumonia

On illness day 12, he became increasingly dyspnoeic, hypoxic and agitated and was intubated and sedated with a midazolam drip. An endotracheal aspirate (ETA) and a further NPS/OPS were collected and sent to the RITM. Vancomycin, 30 mg/kg loading dose followed by 25 mg/kg BD, was commenced with a working diagnosis of severe community-acquired pneumonia due to *Streptococcus pneumoniae* secondary to *Influenza B* infection, plus consideration of COVID-19 pending the ETA result. A complete blood count showed values within the normal range (Table 1). On illness day 13, he continued to be febrile (38.5–40.0 °C) with bibasal crackles. Vital signs were stable with adequate urine output. A chest radiograph was reported as showing worsening of pneumonia (Fig. 5).

**Table 1:** Clinical laboratory results and vital signs

**Hospital day**		**1**	**2**	**3**	**4**	**5**	**6**	**7**	**8**
**Illness day**		**5**	**6**	**7**	**8**	**9**	**10**	**11**	**12**
**Patient 1**	**Ref values**								
Temp (°C)		36.8							
BP (mmHg)		110/80							
Pulse (/min)		84							
Resp (/min)		18							
O2 sat (%)									
NPS/OPS PCR			collected	Influ. B(+) Kl. pneumo (+) S. aureus (+) CoV 229E (+)			SARS-CoV-2 (+)		
NPS/OPS PCR						collected		SARS-CoV-2 (+)	
NPS/OPS PCR*								collected	
Illness day		8	9	10	11	12	13	14	15
**Patient 2**	**Ref values**								
Temp (°C)		38.3	38.8	37.9	38.8	38.1	40	40	40
BP (mmHg)		110/80	110/70	120/80	120/80	130/80	110/70	110/70	110/70
Pulse (/min)		84	86	98	85	94	95	95	95
Resp (/min)		18	22	22	23	38	30	30	30
O2 sat (%)		96% RA		93% 3L O_2_ NP	88% 6L O_2_ FM	91% at 15L O_2_ FM➔ >90% @ 100% Fi0_2_ MV	99% @100% FiO_2_ MV	98% @ 800% FiO_2_ MV	99%@ 80% FiO2 MV
WBC (10^9^/l)	4.0 – 10.0					5.06			9.45
Neutro (%)	55 -65					89.9			85.6
Lymph (%)	25 – 35					7.7			12.2
Mono (%)	3.0 – 8.0					2.4			1.6
Eosino (%)	2.0 – 4.0								0.5
Baso (%)	0 – 1.0								1.6
Hgb (g/l)	120 – 160					143			142
Hct	0.37 – 0.43					0.41			0.41
Plat (x10^9^/l)	150 – 400					188			
NPS/OPS PCR**			collected	Influ. B (+) S. pneumo (+)					
NPS/OPS PCR						collected		SARS-CoV-2 (+)	
ET aspirate PCR						collected			
Blood culture						collected		(-) growth	
**HIV screen**								**non-reactive**	
Date		Jan 25	Jan 26	Jan 27	Jan 28	Jan 29	Jan 30	Jan 31	Feb 1

*NPS/OPS* nasopharyngeal/ oropharyngeal swab, *ETA* endotracheal aspirate

*NPS/OPS—result from RITM was received on February 2 and reported detection of SARS-CoV-2 viral RNA

**NPS/OPS—result from VIDRL was received on February 4 and reported detection of SARS-CoV-2 viral RNA

On illness day 14, increased crepitations in both lung fields were noted. Blood cultures showed no growth after 24 h of incubation. An HIV test was non-reactive. On this day, the RITM reported detection of SARS-CoV-2 viral RNA by real-time PCR from the NPS/OPS taken on illness day 12 and hence the 2nd confirmed COVID-19 infection in the Philippines. This result was later confirmed on February 4 on the initial admission sample sent to VIDRL.

On the morning of illness day 15, the patient remained febrile at 40 °C, with BP 110/70, HR 95, RR 30, SpO_2_ 99% with 80% FiO2, and adequate urine output. However, the patient’s condition deteriorated with the formation of thick sputum and blood clots in the ET tube. Despite frequent suctioning, the patient’s condition deteriorated. He was noted to have laboured breathing followed by a cardiac arrest. Despite several rounds of cardiopulmonary resuscitation, it was not possible to revive the patient. He was thus confirmed as the first COVID-19 death outside of China.

## Discussion and conclusion

This case report describes the first two confirmed cases of COVID-10 in the Philippines and highlights several important clinical and public health issues. Despite both patients being young adults with no significant past medical history, they had very different clinical courses, illustrating how COVID-19 can present with a wide spectrum of disease [8]. Whilst patient 1 had a mild uncomplicated illness consistent with an upper respiratory tract infection and recovery, patient 2 developed a severe pneumonia and died.

One possible explanation for the differing clinical courses is the presence of co-infection. In both patients, the real-time PCR detection panel was reported to be positive for multiple pathogens. The *Staphylococcus aureus* and *Klebsiella pneumoniae* detected in patient 1 most likely represent bacterial colonisation, and it is unclear to what extent her presentation was due to influenza or COVID-19 or both. Patient 2 tested positive for COVID-19, *Influenza B*, and *Streptococcus pneumoniae*, all of which can cause respiratory infection and severe pneumonia. Unfortunately, sputum culture was not possible due to biosafety concerns. It is unclear which pathogen was the leading cause of death, but previous research has shown that outcomes of acute viral respiratory infection are worse if multiple pathogens are present [9]. This highlights the importance of testing for other respiratory pathogens in addition to COVID-19 in order to optimise antimicrobial therapy.

Patient 2 developed increasing dyspnoea on day 11 of illness, similar to the first COVID-19 case in the USA, where mild symptoms were initially reported with progression to pneumonia on day 9 of illness [10]. The median time from illness onset to dyspnoea in a case series in Wuhan was 8 days (range 5–13) [11]. The explanation for patient 2’s worsening condition and development of haemoptysis was progression of pneumonia rather than acute respiratory distress syndrome or pulmonary embolism, but it was not possible to perform a CT scan, additional laboratory tests or an autopsy to further assess this. Although he was treated with broad-spectrum antimicrobials, it is not clear if the outcome would have been better in a high-resource setting. Both patients were treated with oseltamivir in view of positive results for *Influenza B*. Further studies are required to establish the optimal treatment and role of antiviral medication for patients with suspected or confirmed COVID-19 infection.

Our cases contrast with the US case in terms of the relative paucity of lab data and time to receive results. Limited in-house testing was undertaken due to biosafety concerns. In the case of patient 2, the diagnosis of COVID-19 was not made until a day before the patient died. This was because SARS-2-CoV testing was being established in the Philippines at the time that the patients were admitted, and initial samples had to be sent to Australia. Although the delay of diagnosis is unlikely to have altered management, expansion of COVID-19 diagnostics including multiplex panels for other respiratory pathogens is urgently needed for prompt diagnosis of patients for screening of hospital personnel or other contacts.

Three SLH hospital staff who were caring for the patients developed symptoms and themselves became PUIs, but were later discharged following negative SARS-CoV-2 testing and symptom resolution. This highlights the risk of an outbreak in the hospital, or a ‘super-spreader’ scenario, as was observed in other settings during the early stages of the SARS coronavirus infections in 2003 [12]. In the case of SARS, as with COVID-19, SLH managed two cases and was able to contain the infection without further spread [13].

The third confirmed COVID-19 case was announced on February 3 from a sample taken on January 23, also a Chinese national who had travelled from Wuhan. She recovered and returned to China on January 31. Contact tracing has been undertaken of all three patients [14]. Despite travel to several locations in the Philippines whilst experiencing symptoms, as of March 1, there has not been any confirmed local transmission arising from these cases and the number of PUIs has decreased [3]. However, as infection can be mild or subclinical, local transmission cannot be excluded. Increasing the number of laboratories able to perform SARS-CoV-2 testing would allow better surveillance and improve detection of COVID-19 cases.

In conclusion, as of March 1, there have been three confirmed COVID-19 cases in the Philippines including the first death outside of China. No local transmission has been confirmed. Continued vigilance is required to identify new cases.

## Data Availability

N/A

## Abbreviations

COVID-19: Coronavirus disease 2019
nCoV: Novel coronavirus
NPS/OPS: Nasopharyngeal swab/oropharyngeal swab
PCR: Polymerase chain reaction
PUI: Person under observation
RITM: Research Institute for Tropical Medicine
SARS: Severe acute respiratory syndrome
SLH: San Lazaro Hospital
