# Correction to: First COVID-19 infections in the Philippines: a case report

**DOI:** 10.1186/s41182-020-00218-7

**Published:** 2020-05-07

**Authors:** Edna M. Edrada, Edmundo B. Lopez, Jose Benito Villarama, Eumelia P. Salva Villarama, Bren F. Dagoc, Chris Smith, Ana Ria Sayo, Jeffrey A. Verona, Jamie Trifalgar-Arches, Jezreel Lazaro, Ellen Grace M. Balinas, Elizabeth Freda O. Telan, Lynsil Roy, Myvie Galon, Carl Hill N. Florida, Tatsuya Ukawa, Annavi Marie G. Villanueva, Nobuo Saito, Jean Raphael Nepomuceno, Koya Ariyoshi, Celia Carlos, Amalea Dulcene Nicolasora, Rontgene M. Solante

**Affiliations:** 1 San Lazaro Hospital, Manila, Philippines; 2 School of Tropical Medicine and Global Health, Nagasaki University, Nagasaki, Japan; 3 Faculty of Infectious and Tropical Diseases, London School of Hygiene and Tropical Medicine, London, UK; 4 Department of Microbiology, Faculty of Medicine, Oita University, Oita, Japan; 5 Institute of Tropical Medicine, Nagasaki University, Nagasaki, Japan; 6 Research Institute for Tropical Medicine, Alabang, Philippines

**Correction to: Trop Med Health 48, 21 (2020)**

**https://doi.org/10.1186/s41182-020-00203-0**

In the original publication of this article [1], the authors identified an error in the author name of Annavi Marie G. Villanueva.

The incorrect author name is: Annavi Marie G. Villaneuva.

The correct author name is: Annavi Marie G. Villanueva.

The author group has been updated above and the original article [1] has been corrected.
